# Prevalence of Substance Abuse Among Trauma Patients in Rural West Virginia

**DOI:** 10.7759/cureus.36468

**Published:** 2023-03-21

**Authors:** Kanaan Mansoor, Bruno De Souza Goncalves, Hari Vishal Lakhani, Mohammad Tashani, Sharon E Jones, Komal Sodhi, Ellen Thompson, Thomas Dougherty

**Affiliations:** 1 Cardiology, Marshall University Joan C. Edwards School of Medicine, Huntington, USA; 2 Surgery, Marshall University Joan C. Edwards School of Medicine, Huntington, USA; 3 Biomedical Sciences, Marshall University Joan C. Edwards School of Medicine, Huntington, USA; 4 Pharmacology, St. Mary's Medical Center, Huntington, USA; 5 Pathology, St. Mary's Medical Center, Huntington, USA

**Keywords:** west virginia, trauma, substance abuse, drugs, opioid pandemic

## Abstract

Background: Substance abuse poses considerable clinical, economic, and social challenges. West Virginia is hailed as the epicenter of the substance abuse in the United States, the prevalence and pattern of different trauma mechanisms in a rural context or in patients with different forms of substance abuse remain unclear.

Objective: We performed the following analysis to understand the prevalence of substance abuse in patients with different trauma mechanisms in the rural setting with high substance abuse in the West Virginia.

Methods: We performed a cross-sectional retrospective analysis of adult trauma patients (motor vehicle, fall, assault, firearm suicide, brawl/rape and machinery) hospitalized in two tertiary care hospitals in West Virginia between 2006 and 2016. We identified all patients who had a urine drug screen (UDS) test and extracted the data related to the substance and trauma.

Results: Among 8734 patients screened using UDS, 5940 (68.1%) patients were tested positive for the substance. Opiates, alcohol, benzodiazepines, and cannabis were the four most common substances identified in trauma victims. In all instances, the prescribed drug was less than 20%. Fatal outcome was observed in 366 patients in the sample, with 44% (n=162) testing positive for UDS, 12% (n=45) testing positive for only alcohol, and 15% (n=56) testing positive for both alcohol and UDS. Regarding the trauma mechanism, the motor vehicle accident (MVA) was the most prominent with a clear association of substance abuse with fatal outcome.

Conclusion: The most prevalent trauma mechanism was a MVA, with a strong link between drug usage and mortality. Due to the high incidence of positive substance abuse screens, UDS tests may need to be more widely implemented in trauma in the West Virginia region. The findings of this study might help in establishing regional or national policies to reduce acute substance abuse.

## Introduction

Substance abuse has emerged as a major public health crisis, positioning itself as a pandemic with serious health, economic, and social threats to individuals, communities, and governments [1-2]. According to the National Survey on Drug Use and Health (NSDUH), in 2019, 20.4 million people in the US had a substance use problem, with 40.7% of the population having an illicit drug use disorder and 11.8% having both alcohol and illicit drug use disorders. In particular, only 4.2 million received addiction treatment in the previous year, that is, in 2018 [3-5]. Further aggravating the problem is the fact that deaths from drug overdose continue to contribute to overall mortality and the lowering of life expectancy [6]. As a matter of fact, since 1999, rates of deaths secondary to opioids have tripled [7], and, in 2020, the rate was 31% higher than the rate in the previous year [6].

Studies have shown that traumatic injuries are often associated with substance abuse [8-10]; however, the patterns of substance abuse and the associated trauma mechanism have not been fully understood. The emergency department (ED) has evolved into the de facto treatment site for substance abuse presentations; it can serve as public health surveillance resource for substance abuse and traumatic injuries. Patients who abuse drugs are more likely to develop health problems, place a greater load on the healthcare system, and have a higher risk of re-admission [11]. In fact, patients with substance use disorders account for more than half of all emergency department visits in the United States [12-16].

Changes in ED visit patterns have been suggested to reflect drug addiction outbreaks or epidemics [17]. These patterns may be used to figure out how common substance abuse is, how it affects mortality and morbidity, and how to establish policies to address it. In the United States, the prescription drug abuse pandemic is gaining attention, although little is known about its consequences on trauma [18]. Notably, the contribution of substance abuse to complications following traumatic injuries remains unknown.

The prevalence of substance abuse varies dramatically between countries and localities. The substance abuse epidemic in the United States began in the 1990s in places that had endured decades of economic downturn, brain drain, and population shrinkage. A number of variables combined to produce a deplorable situation in which small towns were flooded with prescription opioids [2]. West Virginia is the third-most rural state in the United States, with over half of the population residing in small towns. The Center for Disease Control (CDC) published a report in 2016-2017 that revealed 23 states with significant mortality related to substance abuse epidemics, including West Virginia [7, 19-20]. Indeed, it is currently believed that the United States is facing a pandemic, with West Virginia acting as the epicenter, as shown by the high rate of overdoses and related fatalities. Voelker et al. performed a systematic review of the literature and a semi-structured interview that revealed various strategies employed in West Virginia to keep up with the number of opioid overdoses [20].

It is critical to determine the prevalence and effect of the drug addiction epidemic in order to eradicate it successfully. Rural trauma patients have different injury mechanisms and demographics than those in urban trauma centers. According to the Consumer Product Safety Commission, rural regions account for 60% of all vehicle-related injuries. As a consequence, findings from urban or suburban centers may or may not be applicable in a rural setting [21].

Previous studies have looked at the substance abuse pattern in specific traumatic injuries, but none has done so with such a big sample size and a different trauma mechanism. The primary purpose of this study was to describe the pattern of distribution of substance abuse and the trauma mechanism in a rural setting, since it has already been described that rural populations are more likely to be at high risk of substance use as demonstrated by opioid epidemic of the last decades [22-23]. Additionally, West Virginia, has been impacted by opioids more than any state in the United States and consistently has the highest opioid overdose death rate in the nation [24] . To this end, we conducted a cross-sectional investigation at two tertiary care hospitals with a high-risk rural population in West Virginia with persistent morbidity and mortality due to drug abuse. We have analyzed the prevalence of substance abuse in patients with different trauma mechanisms in the rural setting with high substance abuse in the West Virginia.

## Materials and methods

Study design, location, and population

In this cross-sectional study, we performed a retrospective review of trauma patients who came to the ED of two tertiary care hospitals in West Virginia between 2006 and 2016. Cabell Huntington Hospital and St Mary's Medical Center are two large rural medical centers with a preponderance of multiple drug abuse patients from West Virginia. The approval for this study was received from the Institutional Review Board (IRB) of the Marshall University Joan C. Edwards School of Medicine (IRB No: 956079-3; Effective date: 09/19/2018). After approval, the primary outcome was the positive test for substance abuse, and the secondary outcomes were deposition and mortality.

Inclusion and exclusion criteria and data extraction

To ensure an appropriate selection of patients eligible for the estudy, trained hospital personnel examined patient’s medical records with appropriate confidentiality. The inclusion criteria included all patients who had traumatic injuries and were over 16 years old. Regarding the exclusion criteria, all patients under the age of 16, pregnant females and non-trauma patients were excluded from this study. For the patients included, we collected data on age, gender, substance use, trauma mechanisms (traffic accident, sports accident, aggression, falls of the same level or from a height, blows, and other mechanisms), role of the patient in the incident (whether the patient was a passenger or the driver), and disposition characteristics, which included, death on arrival, admission to the ICU, admission to the floor, transfer to operating room. Shift of the ED (AM, PM), month, year, day of the week were collected to assess the trend of patient presentation. The data were extracted from the electronic medical records. The data were tabulated in Microsoft excel.

Testing

We identified all patients who had a urine drug screen (UDS) test and extracted the type and number of illicit substances that were tested positive. The UDS was conducted using the immunoassay OPI Flex reagent cartridges (Siemens Healthcare Diagnostics, Inc., Newark, DE) in the Siemens Dimension Vista® System (Siemens Healthiness Medical Solutions USA, Inc., Newark, DE). The UDS was done for amphetamines, barbiturates, benzodiazepines, cannabinoids, cocaine, and opiates. A positive alcohol result was defined by the hospitals as a level of ethanol greater than 3 mg/dL.

Statistical analysis

For the data analysis, the presence of substance abuse was dichotomized as positive or negative. Fischer's exact test or chi-square and multinomial regression analysis were performed. Logistic regression was used to estimate odds ratios (OR) and 95% confidence interval (95% CI), Pearson's chi-square was used to assess the distribution of a positive drug screen between sexes, their associated mortality, and disposition. All analyses were performed using SPSS 24.0 for Windows (SPSS, Chicago, IL). p <0.05 was considered significant.

## Results

Demographics

A total of 12924 patients presented to the ED with trauma during the duration of this study. Of these, men were 8941 (69.2%). The mean age was 41.5 ± 16.9 years. In total, 8734 patients with a UDS were tested and 5940 (68.1 %) were found to be positive for substance; with respect to the specific substance, 54.2% (n=3221), 5.2% (n = 311 patients), 4.6% (n = 273), 49% (n= 2912), 7% (n =404), 35.8% (n = 2124), and 64% (n = 3800) were found positive for alcohol, amphetamines, barbiturates, benzodiazepines, cocaine, cannabis, and opiates.

Prescription status and substance abuse

**Figure 1:**
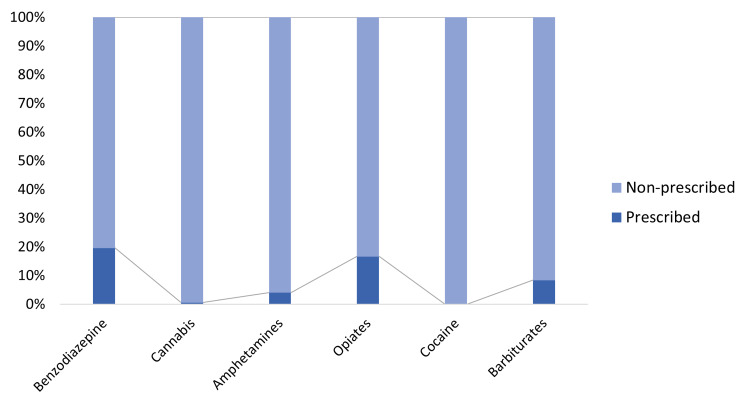
Prescription status of different substances used by the trauma victims.

Figure 1 and Table 1 represent the prescription status of different substances. It can be seen that the percentage of prescribed substances was less than 20% in all cases. Prescribed/non-prescribed cases for benzodiazepine, cannabis, amphetamines, opiates, cocaine, and barbiturates were 572/2340, 12/2118, 13/308, 655/3281, 0/404, and 23/250, respectively.

**Table 1:** Number of substances testing positive on the urine drug screen and distribution based on gender. ∞Drug was either not prescribed or source was unknown; **Other patients not listed in table were either tested negative or had history of abuse or never tested for drug abuse.

Substance	Amount prescribed and not prescribed	Males (n/total%)	Females (n/total%)
Alcohol (n=3221)	-	2551	670
Benzodiazepine (n=2912)	Prescribed (n=572)	403/1953 (20.6%)	169/959 (17.6%)
	N-Prescribed^∞^ (n=2340)	1550/1953 (79.4 %)	790/959 (82.4%)
Cannabis (n=2124)	Prescribed (n=12)	8/1590 (0.5%)	4/534 (0.7%)
	N-Prescribed (n=2118)	1582/1590 (99.5%)	530/534 (99.2%)
Amphetamines (n=311)	Prescribed (n=13)	8/215 (3.7%)	5/96 (5.2%)
	N-Prescribed (n=308)	207/215 (96.3%)	91/96 (94.8%)
(n=3800)	Prescribed (n=655)	426/2574 (16.6%)	228/1226 (18.6%)
	N-Prescribed (n=3281)	2148/2574 (83.4%)	998/1226 (81.4%)
Cocaine (n=404)	Prescribed (n=0)	0 (0%)	0 (0%)
	N-Prescribed (n=404)	296 (100%)	108 (100%)
Barbiturates (n=273)	Prescribed (n=23)	15/164 (9.1%)	8/109 (7.3%)
	N-Prescribed (n=250)	149/164 (90.9%)	101/109 (92.7%)

Sex difference in prescription

The proportion of men was higher in UDS-positive patients, but the results did not achieve statistical significance (67.6% vs 32.4%, p = 0.19). The number of illicit substances and their distribution between men and women are described in Table 2. In all types of substances, the proportion of women was lower, and in terms of prescription status, there was no significant difference between the two groups. Regarding age, the proportion of women in the 16-50 age group who tested positive was 70.6%, and for other age groups, it was 72.9%. It may be noted that 762 women in the age group 16-50 were not tested for drugs compared to 2034 women in the other age group. However, there was a high prevalence of positive UDS (71%, n=1392) amongst women of childbearing age of 16-50 compared to other age groups.

**Table 2:** Distribution of positive UDS amongst females between ages 16 and 50 and all other age groups. UDS, urine drug screen

UDS test	Women between 16 and 50	Women of all other age groups
Positive UDS	1,392	3,280
Negative UDS	580	1,218
Not tested	762	2034

The pattern of trauma mechanism and substance abuse

**Figure 2:**
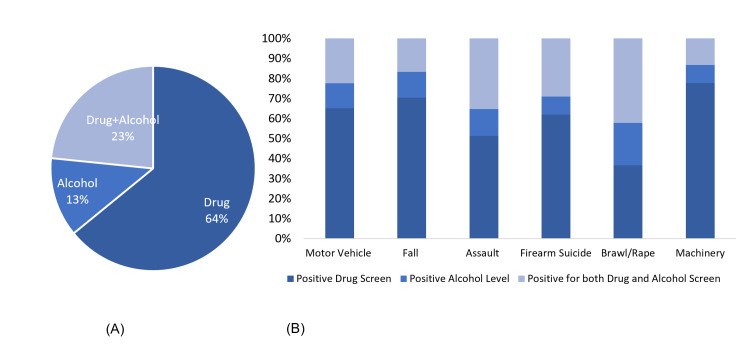
(A) Distribution of drug, alcohol, and drug + alcohol abuse among trauma victims. (B) Trauma mechanism and drug, alcohol, and drug + alcohol abuse.

Figure 2A represents the overall distribution of patients who tested positive for drugs, alcohol, and both. The different mechanisms of trauma are mentioned in Table 3 in the Appendix. Figure 2B shows that the patients tested positive for drugs, alcohol, and both in different trauma mechanisms. It can be seen that in all mechanisms, the number of patients who tested positive for the drug was higher than that who tested positive for alcohol or both alcohol and drug. Indeed, except for brawls and rape, drug use only represented 50% or more cases in the respective categories. Motor vehicle accidents (MVAs) were the main cause of traumatic injuries, followed by falling from height or ladder. In patients with MVAs, 3213 and 618 were positive for drugs and alcohol, respectively. Notably, 1104 were positive for both drugs and alcohol. In patients with suicide or self-injury by firearm, 130/246, 19/246, and 61/246 were positive for drug, alcohol, and both. In terms of blunt, firearm, or unspecified assault, 40-50% of victims were tested positive for drugs while only 6%-18% were tested positive for alcohol. The proportion of patients with assault trauma who tested positive for alcohol and drugs ranged between 21% and 38%.

In cases that presented to the ED due to a MVA and experienced death, there was a significant likelihood that the patient had a positive UDS [OR 0.47(95% CI 0.36-0.62); p = 0.00]. When considering alcohol use in these cases of death from MVA, the probability of having alcohol and a positive UDS was also significant [OR 0.49 (95% CI 0.42-0.57); p = 0.00].

In our sample, 2.4% (n = 305) patients attempted suicide. The distribution of drugs amongst patients who attempted suicide was: 2% amphetamines (n = 6), 4% (n = 11) barbiturates, 37% benzodiazepines (n = 112), 20% cannabis (n = 61), 6% cocaine (n = 18), and 27.2% opiates (n = 83). Concomitant drug use was observed in 3373 patients, notably 35% tested positive for two drugs, 18% tested positive for three drugs, and 3% tested positive for four drugs. A total of 207 patients attempted suicide in the sample, 51% (n= 106) had a positive urine drug test, 26% (n= 54) had a positive urine drug test and alcohol level, and 7% (n=16) had only a positive alcohol level.

There is a significant risk of attempted suicide when using one drug and alcohol [OR 1.54 (95% CI 1.03-2.31); p = 0.04] and when using two drugs and alcohol [OR 2.17 (95% CI 1.4-3.4); p = 0.001]. However, we found that the risk of suicide is not significant when two drugs and ethanol are used concomitantly [OR 1.18 (95% CI 0.65-2.18); p = 0.58].

Deposition and mortality

Among patients who tested positive for UDS, 31% (n=1838) required admission to the floor, 32% (n=1909) required admission to the intensive care unit (ICU), 12% (n=718) required surgical care, and 9% (n=563) of the cases required an observation admission. Fatal results were observed in 5.5% of men (n = 489) and 4.5% of women (n = 177). In patients with positive UDS, fatal outcomes were observed in 221, including 75.1% (n = 166) of men and 24.8% (n = 55) of women without statistically significant outcomes (p = 0.533).

Fatal outcome was observed in 366 patients in the sample, with 44% (n = 162) testing positive for UDS, 12% (n = 45) testing positive for only alcohol, and 15% (n = 56) testing positive for both alcohol and UDS. There was a significant probability that patients with mortality would test positive for one drug on UDS and alcohol [OR 0.69 (95% CI 0.54-0.89); p = 0.004] and tested positive for three drugs and alcohol [OR 0.65 (95% CI 0.47-0.91); p = 0.01]. Interestingly, the risk of death is not significant when two drugs and ethanol are used concomitantly [OR 1.00 (95% CI 0.7-1.43); p = 0.1]; this is perhaps due to the wide variability of substances used in combination.

## Discussion

Trauma is a major cause of fatal outcomes; therefore, identifying factors that affect the propensity to traumatic injuries or mitigate the risk of associated mortality is a high priority. Providing an unsettling perspective from the rural setting, our data highlight the high prevalence of substance abuse in trauma victims and a significant association of substance abuse with the fatal outcome. Most alarmingly, in most cases, substance consumption was not prescribed and the observed substance use pattern and prevalence were different from the typically reported values in trauma patients, mostly aligning with the view that proclaims West Virginia as the epicenter of substance abuse pandemic in the United States. 

In our study, opiates, alcohol, benzodiazepines, and cannabis were the four most common substances identified in trauma victims. In general, 58% of trauma victims were positive for the substance. This prevalence is higher than commonly reported in the literature, because during the years 2006-2016, the drug epidemic was at its peak. The hospitals in Huntington were considered regional trauma centers. For example, a study from the British accident and emergency trauma population, where urine screening was used to monitor drug consumption, revealed a prevalence of 51% drug use, including 13% for cannabinoids, 11% for codeine, 8% for morphine, 6% for amphetamine, 6% for benzodiazepines, 3% for cocaine, 1% for dihydrocodeine, and 1% for methadone [25]. Similarly, in a prospective observational study in trauma patients, the substance was detected in 51 patients (50%), most frequently in men. The substance most commonly detected was alcohol (39%), followed by cannabis (12%) and cocaine (7%), while more than one substance was found in 10 patients (9.8%). The authors concluded that alcohol and/or drug abuse increases the likelihood of recurrent trauma and can shorten the mean trauma-free period among patients without a history of trauma by almost 15 years [26]. Another study on alcohol abuse and illegal substance use patterns in a large cohort of urban trauma patients reported a much lower prevalence. It reported that only 24 % of patients met the criteria for alcohol abuse, and 15% reported using an illegal drug other than marijuana in the past 12 months. Male sex, assaultive injury, substance use, and history of binge drinking were prominent risk factors [27].

In our study, the overall consumption of substances was higher in men than in women, a finding consistent with reported studies [25, 28-29]. However, as stated above, compared to other studies, both prevalence and pattern of substance use were considerably different in our study population, pointing to historical, geographical and locality-based variations. Alcohol is the most frequently consumed substance reported in the literature; however, in our population, opioid consumption was found to be the highest. It seems surprising, particularly because, unlike opioid consumption, alcohol consumption is more common and is considered a norm on certain occasions. Given the high prevalence of drug abuse, it is important to explore a possible association between benzodiazepines, opioids, and tricyclic antidepressants and trauma due to their increased medical consumption [28, 30-31]. In our study, benzodiazepines were the second most common drug found after opiates, which supports the growing trend of the consumption of these drugs, with or without a prescription. Another pharmacologically important drug is amphetamine, which also needs critical scrutiny [32]. From 2003 to 2015, there were 1,292,300 weighted amphetamine-related hospitalizations. In our study, amphetamine was found to be used without any prescription in about 95% of cases. It is logical to recommend the minimization of prescriptions for these substances in the hope of a positive effect on trauma management. The need to regulate the prescribing of these drugs is especially pressing given the recent spike in drug overdoses [33-35]. However, given that benzodiazepines, amphetamines, and opioids were used without a prescription in about 80% of cases in our study, overprescribing these medicines does not seem to be a key influence in their abuse. These observations indicate the need for a more pragmatic and multidimensional strategy in conjunction with auditing and managing medical/non-medical prescriptions.

Opioid usage seems to be a prominent anomaly in our findings; nevertheless, this discovery is predictable given West Virginia's status as the epicenter of the opioid epidemic. It had high overdose rates in 2015, with 41.5 fatalities per 100,000 people, and this soared to 52 deaths per 100,000 people in 2016 [33]. It may be noted that opiate abuse affects 2.5 million Americans with negative impacts on society and the state level [36-37]. In a 17-state analysis from 1999 to 2013 by Leslie et al., total Medicaid costs for patients with opioid use disorder (OUD) tripled from $919 million to $3 billion, respectively [38]. Patients with OUD are more likely to make use of outpatient, ED, and inpatient hospital stays compared to those without OUD [39]. The prevalence of patients abusing prescription opiates has remained relatively stable, according to data from the NSDUH [38, 40]. Our study showed a similar trend, with 17.2% of opiate-positive patients prescribed vs. 82.8% nonprescribed. Essentially, the issue of opioid abuse has significant clinical and economic consequences for patients, healthcare providers, commercial and government agencies, and society as a whole [39]. Therefore, the unique contribution of this study is mainly to delineate the different trauma mechanisms and the respective prevalence of substance abuse, paving the way for more effective mitigation strategies. 

The most common mechanism of trauma in our study was an MVA and, in more than 70% of these cases, the drug or alcohol screening test was positive. Previous studies reported detection of prescription opioids in fatally injured drivers and a significantly higher risk of fatal crash involvement in patients with opioid consumption [41-43]. Furthermore, the use of prescription opioids and alcohol at the same time is related to a 21-fold increase in the probability of being involved in a fatal collision [44]. Therefore, it is recommended that when prescribing opioid analgesics and advising patients, clinicians should keep in mind the harmful impact of opioid analgesics on driving safety [45]. The epidemiological patterns of prescription opioid use in the driver population and the interaction effects between opioids and alcohol on driving safety are not fully ascertained; [46]; our findings provide a valuable perspective, highlighting the high prevalence of substance abuse in rural West Virginia. In fact, the prevalence of substance abuse in victims of MVAs in our study is significantly higher than those reported in other countries. For example, a study from Jordan revealed that alcohol and psychotropic drugs were positive in 36.5% of the cases [47]. Benzodiazepines and barbiturates were among the psychotropic medications found; important, none of the specimens tested positive for illicit cocaine, amphetamines, or cannabis. The findings of this investigation demonstrated the presence of alcohol and psychotropic drugs in road traffic accident victims, showing a link between the use of these substances and the involvement [47]. In particular, the absence of illicit cocaine, amphetamines, or cannabis in this study exemplifies the role of government policies.

One study found that patients who consumed alcohol or drugs had higher severity and a greater prevalence of serious head injuries; however, there was no impact of these substances on the death of wounded patients engaged in car accidents [48]. On the contrary, our study found that there was a high probability that the patient had a positive UDS in the event of a fatal MVA, supporting the view that driving under the influence of alcohol and/or psychoactive substances increases the risk of serious, even fatal MVAs. Essentially, our findings indicate that alcohol and psychotropic drugs are likely risk factors for fatal MVAs [29, 49-50]. The role of substance abuse in the driver's culpability has also been established. It has been reported that drivers who tested positive for alcohol alone, benzodiazepines alone, and combinations of alcohol and THC and alcohol and benzodiazepines were considerably more likely to be at culpability for the accident than the drug-free group [51]. The development, evaluation, and expansion of strategies to prevent alcohol-, drug-, and polysubstance-impaired driving could advance the understanding of drug- and polysubstance-impaired driving and support prevention efforts [52].

Fall (height, ladder, or same level) was another dominant mechanism of trauma in our study, and in more than 60% of cases, it involved the consumption of the substance. When determining the relationship between the consumption of benzodiazepines, opioids, and tricyclic antidepressants and falls, a study showed that variables age, sex, and sensory conditions remain significantly associated, and negatively so with cannabis consumption [53]. In a recent study in Turkey, psychoactive drug treatment was found to be independently associated with the risk of falls [54]. Pratt et al. (2014) discovered that increasing the number or doses of psychoactive medications was related to an increased risk of hospitalization after a fall [53].

Our study revealed a higher percentage of males with positive UDS compared to 30.7% of females. This is consistent with a common theme throughout the literature that males have a higher propensity for traumatic injury than females and a higher incidence of death due to traumatic injury [55]. This is likely due to, at least in part, the increased risk-taking behavior seen in males [56]. Further, by extending the analysis to women trauma victims, we found a high prevalence of substance abuse in women aged 16-50 years. In the setting of illicit drug and alcohol addiction, conception at this age is not safe. Opiates were abused by 30% of the females in this age range. In particular, the misuse of opiates among pregnant women increases the likelihood that newborns have neonatal abstinence syndrome (NAS). Unfortunately, opiate use has resulted in a four-fold increase in the prevalence of NAS between 2003 and 2012, costing the state an estimated $1.5 billion in yearly hospital costs [57-58]. From 2000 to 2013 in West Virginia, the incidence of NAS increased dramatically from 0.5 per 1000 live births to 33.4 per 1000 live births [59-60]. In addition, another study has shown data from 2010 to 2017, and the incidence of NAS in West Virginia increased to 53.5 per 1000 birth [61].

Benzodiazepine was the second most often tested positive substance in 49% of our sample. Most of the use of benzodiazepine in our study was not prescribed, as evidenced by 69% who tested positive. Benzodiazepine use has increased among adults in the United States, with misuse accounting for 20% of all use [62]. Misuse without prescription has been reported to be the most common form of abuse among younger adults, while the older population is more likely to take benzodiazepines more often than prescribed [62-63]. Prescription opioids and stimulant use disorders were most strongly associated with benzodiazepine misuse, indicating a possible synergistic relationship. Therefore, prescription drug monitoring programs are crucial for clinicians to assess whether their patients are abusing various substances and are at risk of misuse of benzodiazepine. Marijuana was another common substance that was tested positive in 35.8% of our study participants. This discovery is not unexpected considering the growing use of recreational marijuana at a time when several states have legalized marijuana. Unfortunately, its abuse is linked to an increased risk of MVAs, particularly fatal accidents [50]. In 2018, 4.7% (12 million) of US residents reported being under the influence of marijuana while driving [52]. Rapid and sensitive testing for law enforcement is therefore crucial to curtailing road traffic accident-related trauma. Amphetamines were tested positive in 5.2% of the sample, with 95% of users having a non-prescribed source, likely reflecting methamphetamine abuse. From 2008 to 2015, US hospitalization rates for amphetamines increased almost four folds, from 55,447 to 206,180 admissions, and were primarily seen in the west [32]. Its use has increased significantly among individuals with opioid use disorder, and this may be attributed to becoming a substitute, allowing a synergistic high or improving functionality while using opioids [64] .

Cocaine was another substance with a modest frequency of 7% in our study. Between 2011 and 2015, the prevalence of cocaine use in the United States increased among adults aged 18-25, women, and those who reported significant alcohol consumption [65]. It is probable that if prescription opiates become less available, drug users may shift their focus to other illegal narcotics. Our research also found that alcohol was present in roughly 54.2% of the patients and that 50% of these patients tested positive for one substance simultaneously, while 16% tested positive for two. Alcohol impairment is a well-known risk factor for trauma because it affects judgment and motor function and the physiological response to injury, increasing the risk of death. Polydrug usage (the use of numerous drugs simultaneously) was prevalent in 53% of the sample. Misuse of polydrug is likely to amplify the effects of these drugs, but it also increases the risk of unsafe driving. Driving under the influence of opioids is risky, with a 72% increase in the risk of a fatal crash when using prescribed opioids. When alcohol is combined with opiates while driving, the danger increases by a factor of 21 [45, 65]. Polydrug use disorders have been more common in the last two decades and are related to poor health, a high chance of imprisonment, increased psychopathology, and suicide attempts and ideation [66-68]. Health professionals should be aware of the increase in polydrug use, and supportive therapy should be the first line of defense until UDS has a conclusive result. Efforts in our society must be maintained to slow the spread of illegal drug and alcohol consumption. Patients who test positive for alcohol or drug misuse are at a higher risk of recidivism and should have the opportunity to break free from these habits [38].

However, the study has several limitations. The main limitations of this study include the impossibility of extrapolating the results to patients who are traumatized and not hospitalized. Furthermore, retrospective collection of some of the variables used in the study through a review of patient medical records. However, patients who were admitted with blood alcohol levels of 0.3 g/L or less have not been considered positive for alcohol. This is because it is the legal limit for novice and professional drivers in our country. Furthermore, we have not adjusted other parameters such as, trauma severity, possibly organ damage after trauma, pathology development, or comma status. A history of substance abuse, a prescription history, psychological disorders, and other related factors can help to reach a specific conclusion. Despite these limitations, this study presents important strengths. To our knowledge, no previous study has investigated the pattern of substance abuse in traumatic injuries with different mechanisms in the rural setting of a developed country. Therefore, we believe that this study provides a new point of view to understand the factors related to trauma incidents and their characteristics. 

## Conclusions

Male patients represented almost 70% of trauma victims with a positive substance test. The MVA was the most prominent trauma mechanism with a clear association of substance abuse with a fatal outcome. Fall trauma was also widespread, demanding specialized prevention measures. Due to the high incidence of positive toxicology tests, these tests may need to be implemented more widely in trauma in the West Virginia region. In general, there were many unique characteristics between the information we have obtained from rural trauma centers in the West Virginia region and the trauma records reported in the literature. Particularly notable was the high incidence of positive opiate tests, which may uniquely reflect the population we serve.

 Rural trauma may have endemic issues related to substance abuse, poverty, and lower degrees of social support when compared to urban areas. The acquisition of objective evidence on drug use patterns from UDS findings allowed us to get insight into current drug use trends in the local community, which we may utilize to develop drug abuse interventions and future drug screening recommendations. This data might be utilized to launch anti-drug-abuse campaigns, build regional or national regulations to regulate acute drug uses, and raise public awareness regarding the high prevalence of substance abuse associated traumas.

## Appendices

**Table 3:** Identified causes for presentation to the ED in patients who were tested with a UDS. ED, emergency department; UDS, urine drug screen

Causes	Distribution in cases	Positive drug screen only	Positive alcohol level only	Positive for both drug and alcohol screen
Motor vehicle accident traffic	6,387	3,213	618	1,104
Fall from height	381	221	28	40
Fall from ladder	265	125	28	32
Assault piercing	255	99	35	83
Firearm suicide/self injury	246	130	19	61
Fall from same level	202	91	24	32
Assault blunt	164	85	15	35
Brawl/rape	156	52	30	60
Machinery	151	70	8	12
Assault firearm	138	70	8	46
Assault unspecified	90	35	17	35
Animal related	64	31	4	11
Falling object	55	32	2	2
Unspecified	35	14	3	10
Explosion	28	20	1	4
Firearm accident	27	17	1	5
Cyclist	20	6	2	5
Burn	13	5	2	5
Hanging	11	11	0	0
Domestic abuse	10	4	2	2
Railway	10	5	1	3
Submersion/drowning	10	5	1	1
Electrocution	9	7	0	0
Aircraft	7	4	0	0
